# Childhood and Adolescence Gender Role Nonconformity and Gender and Sexuality Diversity in Young Adulthood

**DOI:** 10.1001/jamapediatrics.2023.3873

**Published:** 2023-09-25

**Authors:** Jennifer L. Marino, Ashleigh Lin, Cristyn Davies, Melissa Kang, Sarita Bista, S. Rachel Skinner

**Affiliations:** 1 Department of Obstetrics and Gynaecology, Royal Women’s Hospital and University of Melbourne, Parkville, Victoria, Australia; 2 Centre for Adolescent Health, Murdoch Children’s Research Institute, Parkville, Victoria, Australia; 3 Telethon Kids Institute, University of Western Australia, Perth, Western Australia, Australia; 4 Specialty of Child and Adolescent Health, Faculty of Medicine and Health, The University of Sydney, Westmead, New South Wales, Australia; 5 Specialty of General Practice, Faculty of Medicine and Health, The University of Sydney, Westmead, New South Wales, Australia; 6 The Children’s Hospital at Westmead, Westmead, New South Wales, Australia

## Abstract

**Importance:**

Sexuality- and gender-diverse youth experience disproportionate health and social adversity. Accurate early-life indicators are important for development of supportive approaches.

**Objective:**

To examine whether commonly used items measuring childhood conformity to gender roles are associated with sexual orientation in young adulthood.

**Design, Setting, and Participants:**

This single-center, prospective cohort study (the Raine Study) assessed 2868 children of 2900 women who were recruited during pregnancy from August 1, 1989, to April 30, 1992, with follow-up ongoing. The Achenbach System of Empirically Based Assessment (ASEBA) Child Behavior Checklist, Teacher Report Form, and Youth Self-Report tools were used to survey parents, teachers, and youths to examine gender diversity among the participating youths. Parents were followed up at years 5, 8, 10, 14, and 17, adolescents at years 14, 17, and 27, and teachers at years 10 and 14. Data were analyzed from August 1, 2020, to July 31, 2023.

**Exposures:**

Parent and teacher report that a child “behaves like the opposite sex” (gender role behavior), and parent and self-report that a child “wishes to be the opposite sex” (gender role wish), in response to assessment items.

**Main Outcome Measures:**

Year 27 self-reported sexual identity, attraction, and behavior.

**Results:**

Of the 2868 children in the original birth cohort, 1154 (40.2%) participated in the year 27 follow-up, of whom 608 (52.7%) were recorded female at birth and 546 (47.3%) were recorded male at birth. Of these, 582 who were recorded female at birth continued to identify as female (cisgender) (95.7%), and 515 recorded male at birth continued to identify as male (cisgender) (94.3%); 47 (4.1%) did not complete the questionnaire. Of cisgender participants, 76 of 605 women (12.6%) and 52 of 540 men (9.6%) had a diverse sexual identity, 204 of 605 women (33.8%) and 77 of 540 men (14.3%) were same-gender attracted, and 100 of 605 women (18.6%) and 39 of 540 men (7.2%) had ever engaged in same-gender sexual behavior. Across all follow-ups, after adjusting for gender, nonconforming gender role behavior was consistently associated with diverse sexual identity and behavior (adjusted odds ratio [aOR] for identity, 2.8; 95% CI, 1.9-4.2; behavior aOR, 2.4; 95% CI, 1.6-3.5). Self-reported gender role wish was consistently associated with diverse sexual orientation (identity aOR, 2.3; 95% CI, 1.4-3.8; attraction aOR, 1.7; 95% CI, 1.1-2.5; behavior aOR, 1.9; 95% CI, 1.2-2.9).

**Conclusions and Relevance:**

In this cohort study, ASEBA gender role nonconformity was associated with diverse sexual orientation, beginning in early childhood. Findings suggest that the ASEBA measures should not be used to infer sexual orientation or gender diversity in clinical or research settings; asking direct questions may provide more accurate data.

## Key Points

**Question:**

Is gender role nonconformity in childhood and adolescence associated with sexual orientation in young adulthood?

**Findings:**

In this cohort study of 1154 participants, 2 items measuring gender role nonconformity in adolescence, by teacher, parent, and self-report, were significantly associated with diverse sexual orientation in young adulthood. Associations with specific dimensions of sexual orientation and interaction with gender varied by source (self-report, teacher, or parent).

**Meaning:**

These items, which have been used to measure gender diversity in childhood, do not reflect gender identity and, despite these associations, should not be used to infer sexual orientation; more accurate measures should be developed that are asked of children and young people directly.

## Teaser

This cohort study examines whether common measures of childhood conformity to gender roles are associated with sexual orientation and gender diversity in longitudinal, general population birth cohort of Australian young people.

## Introduction

Sexuality- and gender-diverse young people experience disproportionate health and social adversity^1,2^ and barriers to health care access.^3,4,5^ Approximately 5% to 15% of adolescents are sexuality diverse,^6,7,8,9^ and 0.5% to 2% are transgender or gender diverse,^6,7,10,11^ with wide variance due to measurement differences and age, with most estimates in older adolescents (aged ≥15 years). Accurate measurement of sexual orientation and gender diversity in population research is important to better inform our understanding of the relative health and well-being of these minority groups. The Achenbach System of Empirically Based Assessment (ASEBA) Child Behavior Checklist/4-18 (CBCL),^12^ Teacher Report Form (TRF),^13^ and Youth Self-Report (YSR)^14^ have been used to explore gender diversity in children and adolescents. In ASEBA items 110 and 5, respectively, the parent, child or adolescent, or teacher endorses degree of agreement with the statements that the child or adolescent “wishes to be of opposite sex” and “behaves like the opposite sex.” In recent research, these items have been used to measure relationships between gender diversity and autism^15,16,17^ or psychiatric morbidity.^18,19^ However, questions have been raised regarding whether item 110 is a valid indicator of gender diversity in clinical or population settings^15^ and therefore whether it should be used to infer diverse gender identity. These items may measure other dimensions of human experience, such as desire or ability to conform to stereotyped gender roles. Although a previous study found an association between these items in middle childhood and adult same-gender sexual identity, attraction, and behavior,^20^ the association between these items in earlier childhood and adolescence and diverse sexual orientation in adulthood has not been considered. We sought to characterize sexual orientation and gender diversity in a longitudinal, general population birth cohort of Australian young people. We hypothesized that the ASEBA measures of gender role nonconformity in childhood and adolescence would be associated with diverse sexual orientation in young adulthood.

## Methods

### Design and Participants

The Raine Study recruited 2900 pregnant women (generation 1 [Gen1]) in Perth, Australia, between August 1, 1989, and April 30, 1992.^21,22,23^ As part of antenatal demographic data collection, Gen1 mothers reported their own race/ethnicity and that of the father of the child. For both parents, the most common race/ethnicities were Aboriginal (67 mothers [2.3%] and 78 fathers [2.7%]), Chinese (125 mothers [4.4%] and 84 fathers [2.9%]), Indian (75 mothers [2.6%] and 80 fathers [2.8%]), and White (2533 mothers [88.3%] and 2507 fathers [87.4%]). Generation 2 (Gen2) self-report of race/ethnicity has not been ascertained. There were 2868 live births (Gen2), followed up at the ages of 1, 2, 3, 5, 8, 10, 14, 17, 20, 22, and 27 years. Follow-up of Gen1 and Gen2 participants is ongoing. Written informed consent for their own and Gen2 participation was obtained from parents or guardians at each follow-up through 17 years of age and assent from Gen2 participants at 14 and 17 years of age.^24^ At each subsequent follow-up, Gen2 participants provided written informed consent. Teachers provided written informed consent for participation at the year 10 and 14 follow-ups. The Raine Study and each follow-up have institutional human research ethics approval, most recently from the University of Western Australia Human Research Ethics Committee. This specific project was approved through Curtin University.^25^ The current prospective cohort study is reported according to the Strengthening the Reporting of Observational Studies in Epidemiology (STROBE) reporting guideline.^26^

### Language

Language used to describe sexuality- and gender-diverse populations is constantly changing. Some terms used in the Raine Study, including the since-updated CBCL, YSR, and TRF, are no longer considered culturally safe or accurate to capture sexual orientation and gender diversity: *opposite sex* assumes binary gender rather than a continuum; *sex* is used when *gender* is now most appropriate; gender and sexual orientation are measured in the same item; and *male, boy,* and *man* are used interchangeably for both gender and sex, as are *female, girl,* and *woman*.^27,28^ Measures to capture sexual orientation and gender diversity need review and updating in partnership with these communities, with the use of qualitative research methods.^29^

Reflexivity statements evidence critical reflection on researcher positionality. Such statements are not common in quantitative research but are standard and best practice in qualitative research^30,31^ and research with minority groups.^29^ Four authors have lived experience as sexuality or gender diverse, and none belong to the Millennial generation (born 1981-1996).

### Gender Role Conformity in Childhood and Adolescence

Gender role conformity was based on the ASEBA items “behaves like the opposite sex” (gender role behavior) and “wishes to be of opposite sex” (gender role wish). The CBCL was completed by the primary caregiver at 5-, 8-, 10-, 14-, and 17-year follow-ups, the TRF was completed by a teacher at 10- and 14-year follow-ups, and the YSR was completed by Gen2 participants at 14- and 17-year follow-ups. The TRF asks about gender role behavior, the YSR asks about gender role wish, and the CBCL asks about both.

### Sexual Orientation and Gender Identity in Adulthood

Sexual identity, attraction, and behavior were measured at year 27. Identity was measured by asking, “What do you identify as,” with response options “heterosexual,” “gay/lesbian,” “bisexual,” “not sure,” and “other–please specify.” Attraction was measured by asking, “Which of these statements best describes you,” with response options “I have felt attracted… “…only to females, never to males,” “…more often to females and at least once to a male,” “I am about equally attracted to females and males,” “…more often to males and at least once to a female,” “…only to males, never to females,” and “I have never felt attracted to anyone at all.” Behavior was based on responses to 3 items: gender of primary partner if applicable (“male,” “female,” “other–please specify”), gender(s) of sexual partner(s) over the past year, and gender(s) of sexual partner(s) over the lifetime (both “male only,” “female only,” “male and female”). Gender identity was determined by comparing sex recorded at birth to a year 27 follow-up item, which asked, “Do you identify as:” with response options “female,” “male,” “transgender female,” “transgender male,” “nonbinary,” and “other–please specify.”

### Statistical Analysis

Categorical variables are summarized with numbers (percentages), normally distributed continuous variables with means (SDs), and nonnormally distributed continuous variables with medians (IQRs and ranges). Distribution differences were tested using the Pearson χ^2^ or, for small cell sizes, Fisher exact test, with a 2-sided α = .05. Follow-ups were classified into childhood (5, 8, or 10 years) or adolescent (14 or 17 years), except for teacher-report data, which included only 10- and 14-year follow-ups. Participants were counted as having nonconforming gender role behavior or wish if the response to the item was “somewhat or sometimes true” or “often or very true” and as conforming if the response was “not true (as far as you know).”

To overcome the sparse population in some sexual orientation categories, sexual identity was binarized as “heterosexual” or “diverse,” sexual attraction as “heterosexual only” or (any) “same-gender sexual attraction,” and sexual behavior as “heterosexual only” or (any) “same-gender sexual behavior.” Logistic regression was used to calculate measures of association (odds ratios [ORs] and 95% CIs) after adjusting for gender.

When all dimensions of sexual orientation were considered together, it was possible to examine associations separately for cisgender male and female participants. Comparisons were made between those who were heterosexual in all 3 dimensions of orientation (identity, attraction, and behavior) and all participants endorsing any diverse sexual identity, same-gender sexual attraction, or same-gender sexual behavior (diverse sexual orientation). Interaction was assessed by Wald test on interaction terms between gender and ASEBA measure, with a 2-sided test α = .20, a less stringent standard than interaction models are typically under.

Demographic comparisons with population-based data used 2016 Australian Census data for persons aged 25 to 29 years living in Western Australia.^32^ Analyses were conducted using Stata software, version 15.0 (StataCorp LLC).

## Results

### Demographic Characteristics

Demographic characteristics of the Gen2 Raine Study participants during childhood and adolescence are shown in Table 1. The proportion of participants recorded as male at birth did not change with attrition through adolescence, but family income increased. At year 27, most participants had completed some postsecondary education, were partnered, and worked outside the home (Table 2). Compared with contemporaneous Western Australian 25- to 29-year-olds, Gen2 participants of the Raine Study were more likely to be employed full time (658 [57.0%] vs 91 138 [49.4%]), but weekly personal incomes were similar (eTable 1 in Supplement 1).

**Table 1.** Demographic Characteristics at Each Follow-Up of All Generation 2 Participants and of Those Who Participated in Year 27 Follow-Up of the Raine Study^d^

Characteristic	Year 5 (1995-1999)	Year 8 (1998-2000)	Year 10 (2000-2003)	Year 14 (2003-2006)	Year 17 (2004-2009)
**Total**	**2200**	**2113**	**2024**	**1554 Dyads, 245 P only, 51 A only**	**1098 Dyads, 302 P only, 138 A only**
Sex recorded at birth					
Female	1066 (48.4)	1024 (48.5)	975 (48.2)	880 (48.9),^a^ 783 (48.8)^b^	692 (49.0),^a^ 624 (50.0)^b^
Male	1134 (51.6)	1089 (51.5)	1049 (51.8)	919 (51.1),^a^ 822 (51.2)^b^	721 (51.0),^a^ 625 (50.0)^b^
Age, mean (SD), y	5.94 (0.21)	8.09 (0.35)	10.60 (0.20)	14.11 (0.21),^a^ 14.10 (0.19)^b^	17.03 (0.23),^a^ 17.02 (0.24)^b^
Family structure^a^					
Single parent	414 (18.8)	414 (19.6)	422 (20.8)	412 (22.9)	351 (24.8)
Couple	1773 (80.6)	1696 (80.3)	1598 (79.0)	1385 (77.0)	1059 (75.0)
Other or unknown	13 (0.6)	3 (0.1)	4 (0.2)	2 (0.1)	3 (0.2)
Annual family income, A$^a^^,^^c^					
<25 000	639 (29.1)	437 (20.7)	426 (21.0)	230 (12.8)	108 (7.6)
25 000-40 000	561 (25.5)	492 (23.3)	371 (18.3)	303 (16.8)	148 (10.5)
>40 000	905 (41.1)	NA	NA	NA	NA
40 000-60 000	NA	504 (23.9)	477 (22.1)	357 (19.8)	209 (14.8)
>60 000	NA	615 (29.1)	734 (36.3)	NA	NA
60 000-104 000	NA	NA	NA	557 (31.0)	451 (31.9)
>104 000	NA	NA	NA	321 (17.8)	459 (32.5)
Missing or unknown	95 (4.3)	65 (3.1)	46 (2.3)	31 (1.7)	38 (2.7)
**Attended year 27 follow-up**	**1056 (48.0)**	**1058 (50.1)**	**1061 (52.4)**	**914 Dyads (58.8), 94 P only (38.3), 26 A only (51.0)**	**769 Dyads (70.0), 105 P only (34.8), 86 A only (62.3)**
Sex recorded at birth					
Female	553 (52.4)	554 (52.3)	552 (52.0)	531 (52.7),^a^ 492 (52.3)^b^	448 (51.3),^a^ 446 (52.2)^b^
Male	503 (47.6)	505 (47.7)	509 (48.0)	477 (47.3),^a^ 448 (47.7)^b^	426 (48.7),^a^ 409 (47.8)^b^
Age, mean (SD), y	5.93 (0.19)	8.07 (0.34)	10.59 (0.18)	14.11 (0.18),^a^ 14.10 (0.17)^b^	17.02 (0.21),^a^ 17.02 (0.22)^b^
Family structure^a^					
Single parent	165 (15.6)	176 (16.6)	184 (17.3)	188 (18.7)	194 (22.2)
Couple	886 (83.9)	882 (83.3)	874 (82.4)	820 (81.3)	679 (77.7)
Other or unknown	5 (0.5)	1 (0.1)	3 (0.3)	0	1 (0.1)
Annual family income, A$^a^^,^^c^					
<25 000	253 (24.0)	178 (16.8)	181 (17.1)	101 (10.0)	57 (6.5)
25 000-40 000	261 (24.7)	227 (21.5)	193 (18.2)	151 (15.0)	78 (8.9)
>40 000	510 (48.3)	NA	NA	NA	NA
40 000-60 000	NA	263 (24.8)	244 (23.0)	191 (18.9)	122 (14.0)
>60 000	NA	361 (34.1)	426 (40.2)	NA	NA
60 000-104 000	NA	NA	NA	343 (34.0)	289 (33.1)
>104 000	NA	NA	NA	209 (20.7)	309 (35.3)
Missing or unknown	32 (3.0)	29 (2.7)	17 (1.6)	13 (1.3)	19 (2.2)

Abbreviations: A, adolescent; NA, not applicable; P, parent.

^a^ Some adolescents participated independently. This is a parent-reported measure; percentage uses denominator of all parent-reported measures.

^b^ Adolescent-reported measure; percentage uses denominator of all adolescent-reported measures.

^c^ Categories of income in data collection changed over time to reflect income distribution in Western Australia. All figures are Australian dollars at the time of data collection. (To convert to US dollars as of August 26, 2023, multiply by 0.6417.)

^d^ Data are presented as number (percentage) of participants unless otherwise indicated.

**Table 2.** Characteristics of Generation 2 Participants at Year 27 Follow-up^a^

Characteristic	Year 27 (2017-2020)
Total	1154 (100)
Sex recorded at birth	
Female	608 (52.7)
Male	546 (47.3)
Age, mean (SD), y	26.7 (0.4)
Educational level	
Less than secondary school	2 (0.2)
Secondary school	250 (21.7)
Apprentice, TAFE, or other training course	364 (31.5)
Tertiary degree	404 (35.0)
Posttertiary degree	128 (11.1)
Missing or not stated	6 (0.5)
Relationship status	
Single, not in a relationship	367 (31.8)
In a relationship, not living together	185 (16.0)
In a relationship, living together	420 (36.4)
Married	138 (12.0)
Missing	44 (3.8)
Employment	
Employed full time	658 (57.0)
Employed part time, casually, or unpaid	283 (24.5)
Employed but on long-term or maternity leave	46 (4.0)
Home duties or carer duties	9 (0.8)
Self-employed, contractor, or freelancer	20 (1.7)
In study or training only	34 (2.9)
Unemployed or out of labor force and not in study or training	31 (2.7)
Other or unspecified	18 (1.6)
Missing	55 (4.8)
Annual household income, A$^b^	
<78 000	337 (29.2)
78 000-<130 000	278 (24.1)
130 000-<182 000	224 (19.4)
>182 000	128 (11.1)
Don’t know	178 (15.4)
Missing	9 (0.8)

Abbreviation: TAFE, technical and further education.

^a^ Data are presented as number (percentage) of participants unless otherwise indicated.

^b^ All figures are Australian dollars at the time of data collection. (To convert to US dollars as of August 26, 2023, multiply by 0.6417.)

### Gender Identity in Adulthood

Of the original birth cohort (N = 2868), 1154 individuals (40.2%) participated in the year 27 follow-up, of whom 608 (52.7%) were recorded female and 546 (47.3%) male at birth, a change from earlier follow-ups, where slightly more participants were recorded male. Of these 1154 individuals, 582 who were recorded female at birth continued to identify as female (cisgender) (95.7%); 515 recorded male at birth continued to identify as male (cisgender) (94.3%); and 47 (4.1%) did not complete the questionnaire. The remaining 10 (0.9%) whose gender differed from sex recorded at birth identified as “male,” “female,” “transgender female,” “nonbinary,” or “other.” There were too few gender-diverse participants for further analysis.

### Sexual Orientation in Adulthood Among Cisgender Participants

For cisgender participants, dimensions of sexual orientation are presented in Table 3. Diverse sexual identity did not significantly differ between women and men (76 of 604 women [12.6%] vs 52 of 540 men [9.6%], *P* = .40), but the distribution of identities differed. Within the diverse group, men were more likely to identify as gay than women as lesbian (21 of 52 men [40.4%] vs 14 of 76 women [18.4%], *P* = .009). More women than men expressed same-gender attraction (204 of 604 women [33.8%] vs 77 of 540 men [14.3%], *P* < .001) or behavior (100 of 504 women [16.6%] vs 39 of 540 men [7.2%]; *P* < .001). More than twice as many women as men reported any sexuality diversity (230 of 604 women [38.1%] vs 92 of 540 men [17.0%], *P* < .001).

**Table 3.** Sexual Orientation in Adulthood (Age 27 Years) Among Cisgender Participants

Sexual orientation	Cisgender participants, No (%)	
	Female (n = 604)	Male (n = 540)
Sexual identity		
Heterosexual^a^	501 (82.9)	458 (84.8)
Gay or lesbian	14 (2.3)	21 (3.9)
Bisexual	29 (4.8)	13 (2.4)
Not sure	24 (4.0)	16 (3.0)
Other, specify^b^	9 (1.5)	2 (0.4)
Missing^c^	27 (4.5)	30 (5.5)
Any nonheterosexual identity	76 (12.6)	52 (9.6)
Sexual attraction		
Only females, never males	6 (1.0)	435 (80.6)
More often females, at least 1 male	12 (2.0)	49 (9.1)
Equally male and female	21 (3.5)	5 (0.9)
More often males, at least 1 female	165 (27.3)	8 (1.5)
Only males, never females	376 (62.2)	15 (2.8)
Never attracted to anyone	2 (0.3)	3 (0.6)
Missing	22 (3.6)	25 (4.6)
Any same-gender sexual attraction	204 (33.8)	77 (14.3)
Primary partner		
Male	393 (65.1)	12 (2.2)
Female	17 (2.8)	311 (58.0)
Other, specify	1 (0.2)	0
Not applicable (no partner)	152 (25.2)	181 (33.5)
Missing	41 (6.8)	36 (6.7)
Sexual partners, last 12 mo		
Male	509 (84.3)	23 (4.3)
Female	18 (3.0)	448 (83.0)
Both	14 (2.3)	4 (0.7)
Never partnered	35 (5.8)	37 (6.8)
Missing	28 (4.6)	28 (5.2)
Sexual partners lifetime		
Male	443 (73.3)	18 (3.3)
Female	3 (0.5)	436 (80.7)
Both	95 (15.7)	21 (3.9)
Never partnered	35 (5.8)	37 (6.8)
Missing	28 (4.6)	28 (5.2)
Any same-gender sexual behavior	100 (18.6)	39 (7.2)
Any diverse sexual orientation	230 (38.1)	92 (17.0)

^a^ Included those who specified “straight,” “heterosexual but gay for Johnny Depp,” cisgender male participants who specified “female only,” and cisgender female participants who specified “male only” (n = 6).

^b^ Included “pansexual,” “pansexual; gray asexual and gray aromantic,” “asexual,” “bicurious,” “none–love is the only thing I identify with,” “normal,” “queer,” “fluid/queer,” and no text supplied.

^c^ Included 2 participants who specified “Apache attack helicopter,” an established mischief response usually presented to gender rather than sexual identity items.

### Gender Role Behavior and Wish in Childhood and Adolescence and Sexuality in Adulthood

At all follow-ups, nonconforming behavior was more common than nonconforming wish (Table 4). After adjusting for gender, nonconforming behavior in childhood and adolescence, irrespective of reporting source, was consistently associated with diverse sexual identity and behavior in adulthood. Patterns were less clear for associations with adult same-gender sexual attraction. Parent-reported nonconforming behavior in adolescence and teacher-reported nonconforming behavior in childhood were associated with same-gender sexual attraction in adulthood. Although point estimates for ORs for both reporting sources at all time points were above 1, only parent-reported nonconforming wish in childhood and self-reported nonconforming wish in adolescence were statistically significantly associated with all dimensions of adult sexual orientation. Similarly, parent-reported nonconforming wish in adolescence was associated only with nonheterosexual behavior, not other dimensions, likely reflecting the much rarer endorsement of nonconforming wish by parents than by adolescents.

**Table 4.** ASEBA Gender Role Measures in Childhood and Adolescence and Dimensions of Sexual Orientation in Adulthood (Age of 27 Years) Among Cisgender Participants

ASEBA gender role measures in childhood and adolescence	Diverse gender identity, No.	Sexual identity at 27 y			Sexual attraction at 27 y^a^			Sexual behavior at 27 y		
		No. (%)		aOR (95% CI)^b^	No. (%)		aOR (95% CI)^b^	No. (%)		aOR, (95% CI)^b^
		Heterosexual	Diverse		Heterosexual only	Same gender		Heterosexual only	Same gender	
All	10	959 (100)	128 (100)	NA	811 (100)	281 (100)	NA	887 (100)	139 (100)	NA
Behaves like opposite sex (ASEBA gender role behavior)										
Parent report										
Year 5	2	99 (11.4)	25 (22.1)	2.2 (1.3-3.6)	84 (11.3)	42 (17.4)	1.6 (1.0-2.4)	87 (11.0)	25 (20.7)	2.1 (1.3-3.5)
Year 8	1	65 (7.5)	25 (21.6)	3.3 (2.0-5.5)	59 (8.1)	33 (12.8)	1.4 (0.9-2.3)	58 (7.3)	25 (19.7)	2.9 (1.8-4.9)
Year 10	1	37 (4.2)	16 (13.6)	3.5 (1.9-6.5)	33 (4.4)	21 (8.2)	1.7 (0.9-3.0)	33 (4.1)	14 (11.2)	2.6 (1.3-5.0)
Any childhood follow-up	2	146 (15.6)	40 (32.5)	2.5 (1.6-3.8)	128 (16.2)	61 (22.3)	1.4 (1.0-1.9)	131 (15.2)	40 (29.4)	2.2 (1.5-3.3)
Year 14	1	19 (2.3)	12 (11.0)	5.2 (2.5-11)	18 (2.5)	13 (5.4)	2.0 (0.9-4.2)	19 (2.5)	11 (8.9)	3.7 (1.7-8.1)
Year 17	1	12 (1.6)	9 (9.8)	6.3 (2.5-16)	9 (1.5)	12 (5.5)	2.9 (1.2-7.0)	10 (1.5)	9 (8.3)	4.8 (1.9-12)
Any adolescent follow-up	1	30 (3.4)	16 (14.0)	4.3 (2.2-8.1)	26 (3.5)	20 (7.8)	2.0 (1.1-3.7)	28 (3.4)	16 (12.7)	3.6 (1.9-7.0)
Teacher report										
Year 10	0	7 (0.9)	6 (5.4)	6.9 (2.3-21)	7 (1.0)	7 (2.9)	3.5 (1.2-10.5)	6 (0.8)	6 (5.0)	7.0 (2.2-23)
Year 14	0	8 (1.4)	6 (8.5)	6.5 (2.2-19)	8 (1.7)	6 (3.6)	2.3 (0.7-6.9)	10 (1.9)	4 (5.5)	2.9 (0.9-9.8)
Any follow-up	0	15 (1.7)	12 (10.1)	6.7 (3.0-15)	15 (2.0)	13 (5.0)	2.9 (1.3-6.3)	16 (2.0)	10 (7.8)	4.4 (1.9-10)
Either source										
Any follow-up	2	171 (18.1)	49 (39.2)	2.8 (1.9-4.2)	151 (18.9)	72 (26.0)	1.4 (1.0-1.9)	154 (17.6)	48 (35.0)	2.4 (1.6-3.5)
Wishes to be opposite sex (ASEBA gender role wish)										
Parent report										
Year 5	0	13 (1.5)	8 (7.0)	4.8 (1.9-12)	10 (1.3)	11 (4.4)	3.0 (1.2-7.3)	8 (1.0)	11 (8.7)	9.0 (3.5-23)
Year 8	0	10 (1.2)	9 (7.8)	7.2 (2.8-18)	9 (1.2)	10 (3.9)	3.1 (1.2-8.0)	6 (0.8)	10 (7.8)	11.3 (3.9-32)
Year 10	0	4 (0.4)	5 (4.2)	9.1 (2.4-34)	4 (0.5)	5 (2.0)	2.7 (0.7-10.3)	2 (0.2)	9 (8.1)	16.3 (3.2-83)
Any childhood follow-up	0	23 (2.5)	14 (11.4)	4.8 (2.4-9.7)	20 (2.5)	17 (6.2)	2.3 (1.2-4.5)	14 (1.6)	18 (13.2)	9.0 (4.3-19)
Year 14	0	4 (0.5)	2 (1.8)	3.6 (0.7-20)	3 (0.4)	3 (1.2)	2.3 (0.4-11.6)	4 (0.5)	2 (1.6)	2.6 (0.5-14)
Year 17	0	2 (0.3)	2 (2.2)	7.4 (1.0-54)	1 (0.2)	3 (1.4)	5.4 (0.6-52)	1 (0.2)	3 (2.8)	13.6 (1.4-133)
Any adolescent follow-up	0	6 (0.7)	3 (2.6)	3.5 (0.9-14)	4 (0.5)	5 (2.0)	2.6 (0.7-10)	5 (0.6)	4 (3.2)	4.0 (1.1-15)
Self-report										
Year 14	0	33 (4.2)	7 (6.9)	1.5 (0.6-3.6)	28 (4.2)	12 (5.4)	1.0 (0.5-2.0)	28 (3.9)	9 (8.1)	1.8 (0.8-3.9)
Year 17	2	59 (8.4)	20 (21.1)	2.8 (1.5-5.0)	44 (7.4)	35 (16.6)	2.1 (1.3-3.4)	52 (8.0)	20 (19.2)	2.4 (1.4-4.3)
Any follow-up	2	81 (9.6)	23 (20.7)	2.3 (1.4-3.8)	63 (7.8)	41 (14.6)	1.7 (1.1-2.5)	73 (9.4)	24 (20.0)	1.9 (1.2-2.9)
Either source										
Any follow-up	2	101 (10.7)	33 (25.6)	2.7 (1.7-4.3)	81 (10.1)	52 (18.8)	1.7 (1.2-2.5)	86 (9.8)	35 (25.5)	2.7 (1.7-4.3)

Abbreviations: aOR: adjusted odds ratio, ASEBA: Achenbach System of Empirically Based Assessment.

^a^ Participants never attracted to anyone (n = 5) are omitted.

^b^ Adjusted for gender. The aOR estimates greater than 10 are reported in whole units to limit overprecision.

Considering all dimensions of sexual orientation together, nonconforming behavior was consistently associated with diverse sexual orientation (Table 5). Parents were more likely to report nonconforming behavior than teachers, and within parent reports, nonconforming behavior was more common during childhood than adolescence. Gender modified associations between parent-reported nonconforming behavior and sexual orientation, with statistically significant associations only among boys. This gender interaction was not seen in adolescence or in teacher reports, but this may reflect poor power due to lower reporting rates. When categories were collapsed across reporting source and follow-ups, associations between gender role behavior and sexual orientation were consistently stronger among boys. Similarly, nonconforming gender role wish was uncommon, parents were much less likely to endorse nonconforming wish than were adolescents, and nonconforming wish was less common among boys than girls across all reporting sources. Interactions between gender and nonconforming wish could not be examined in parent-reported data because of empty strata. In self-report at 17 years and when categories were collapsed across follow-ups, nonconforming wish was associated with diverse sexual orientation, without evidence of interaction by gender. When categories were collapsed across sources and follow-ups, nonconforming wish was related to diverse sexual orientation among both male and female participants; there was also an interaction with gender, and although the higher bound of the CI in girls slightly overlapped the lower CI in boys, the association of sexual orientation with gender role wish appeared to be stronger in boys. The probability of participation in the year 27 follow-up was consistently and positively associated with the probability of participation in previous follow-ups but was not associated with the probabilities of completing ASEBA instruments or gender role items or of endorsing nonconforming gender role behavior or wish at those follow-ups (eTable 2 in Supplement 1).

**Table 5.** ASEBA Gender Measures in Childhood and Adolescence And Diverse Sexual Orientation (Binarized as Heterosexual Only or Any Sexually Diverse Identity, Same-Gender Sexual Attraction, or Same-Gender Sexual Behavior) in Adulthood by Gender Among Cisgender Participants

ASEBA gender role measures in childhood and adolescence	*P* value for interaction between gender and measure	Cisgender female (n = 582)			Cisgender male (n = 516)			Combined (n = 1098) aOR (95% CI)^a^^,^^b^
		Heterosexual only, No. (%) (n = 352)	Sexuality diverse, No. (%) (n = 230)	OR (95% CI)^a^	Heterosexual only, No. (%) (n = 422)	Sexuality diverse, No. (%) (n = 94)	OR (95% CI)^a^	
Behaves like opposite sex (ASEBA gender role behavior)								
Parent report								
Year 5	.14	43 (13.2)	30 (15.5)	1.2 (0.7-2.0)	37 (9.6)	16 (19.0)	2.2 (1.2-4.2)	NA
Year 8	.04	36 (11.5)	26 (12.5)	1.1 (0.6-1.9)	19 (4.9)	11 (13.1)	2.9 (1.3-6.4)	NA
Year 10	.001	25 (7.8)	13 (6.3)	0.8 (0.4-1.6)	7 (1.8)	9 (10.5)	6.5 (2.3-18)	NA
Any childhood follow-up	.003	71 (20.2)	44 (19.1)	0.9 (0.6-1.4)	50 (11.8)	24 (26.1)	2.6 (1.5-4.6)	NA
Year 14	.48	10 (3.2)	10 (5.0)	NA	7 (1.9)	4 (5.0)	NA	1.9 (0.9-3.1)
Year 17	.41	6 (2.4)	12 (7.0)	NA	1 (0.3)	2 (2.8)	NA	3.7 (1.5-9.5)
Any adolescent follow-up	.29	15 (4.3)	17 (7.5)	NA	8 (1.9)	6 (6.5)	NA	2.2 (1.2-4.1)
Teacher report								
Year 10	.31	3 (1.0)	3 (1.5)	NA	4 (1.1)	4 (4.8)	NA	4.6 (1.1-19)
Year 14	.27	4 (1.9)	4 (2.9)	NA	3 (1.2)	3 (5.8)	NA	5.1 (1.0-26)
Any follow-up	.13	7 (2.0)	7 (3.0)	1.5 (0.5-4.5)	7 (1.7)	7 (7.6)	4.9 (1.7-14)	NA
Either source								
Any follow-up	.003	82 (23.3)	54 (23.5)	1.0 (0.7-1.5)	59 (14.0)	28 (30.4)	2.7 (1.6-4.6)	NA
Wishes to be opposite sex (ASEBA gender role wish)								
Parent report								
Year 5	.02	8 (2.5)	7 (3.5)	1.4 (0.5-4.0)	1 (0.3)	5 (6.0)	24 (2.8-213)	NA
Year 8	.26	5 (1.6)	7 (3.3)	NA	3 (0.8)	4 (4.8)	NA	3.3 (1.3-8.5)
Year 10	Inestimable	2 (0.6)	6 (2.9)	4.7 (0.9-24)	0	1 (1.2)	Inestimable	6.4 (1.3-32)
Any childhood follow-up	.02	12 (3.4)	13 (5.7)	1.7 (0.8-3.8)	4 (0.9)	8 (8.7)	10 (2.9-34)	NA
Year 14	Inestimable	2 (0.6)	3 (1.5)	2.3 (0.4-14.0)	1 (0.3)	0	Inestimable	1.8 (0.4-9.4)
Year 17	Inestimable	1 (0.4)	3 (1.8)	4.5 (0.5-44.1)	0	0	Inestimable	4.5 (0.5-44)
Any adolescent follow-up	Inestimable	3 (0.9)	5 (2.2)	2.6 (0.6-10.9)	1 (0.2)	0	Inestimable	2.2 (0.6-8.3)
Self-report								
Year 14	.41	21 (7.3)	12 (6.6)	NA	5 (1.4)	2 (2.7)	NA	1.0 (0.5-2.0)
Year 17	.90	23 (9.0)	34 (20.2)	NA	14 (4.50	8 (11.4)	NA	2.6 (1.6-4.2)
Any follow-up	.26	38 (10.8)	39 (17.0)	NA	17 (4.0)	10 (10.9)	NA	1.9 (1.3-2.9)
Either source								
Any follow-up	.02	48 (13.6)	47 (20.4)	1.6 (1.0-2.5)	21 (5.0)	17 (18.5)	4.3 (2.2-8.6)	NA

Abbreviations: aOR, adjusted odds ratio; ASEBA, Achenbach System of Empirically Based Assessment; NA, not applicable.

^a^ Odds ratio estimates greater than 10 are reported in whole units to limit overprecision. Combined measures of association are presented where there was no evidence of interaction with gender.

^b^ Odds ratio adjusted for gender.

## Discussion

In this longitudinal cohort study of young Australians, from 5 to 17 years of age, the ASEBA items 5 and 110 (“behaves like the opposite sex” and “wishes to be the opposite sex,” respectively) were associated with diverse sexual orientation for all measured dimensions of sexual orientation (identity, attraction, and behavior). This finding contrasts with the only other population-based prospective study using these measures, from Steensma and colleagues,^20^ which found that parent-reported nonconforming behavior and wish measured at a mean age of 7.5 years were related to dimensions of sexual orientation at 30 years of age in both men and women, but only to homosexuality, not bisexuality. We could not examine bisexuality separately from exclusive homosexuality because of small numbers. When power allowed formal analysis, we found that gender modified associations with nonconforming behavior and wish, with stronger associations among boys. This difference may be because we examined gender nonconformity at older ages than Steensma et al.^20^

We also found that adolescents were more likely to report nonconforming wish than parents were to report this of their children, in either childhood or adolescence, and that parents were more likely to report nonconforming behavior than teachers. Low statistical power may account for weak or absent associations, but it is also likely that the source matters—parents have more opportunity to observe their children in unstaged moments than do teachers, and adolescents may be franker about their feelings on a survey than they are with their parents. If adolescence is a time of gender intensification, wherein social pressure to conform to gendered role behavior increases,^33^ then adolescents may feel particularly vulnerable in disclosing nonconforming gender role wish and may not share this with parents. This possibility supports recent recommendations that in studies of sexual orientation and gender identity, adolescents should report for themselves wherever possible.^10,34^

Understanding sexuality and gender diversity is important because of the stigma and discrimination experienced by many sexuality- and gender-diverse young people. Disparities relative to cisgender, heterosexual peers include poorer mental health, such as depression,^35,36^ anxiety,^37^ self-harm,^36,38^ suicidal ideation and attempts,^1,38,39^ and posttraumatic stress disorder,^40,41^ with transgender young people at highest risk.^42,43^ Stigma, exclusion, and minority stress are thought to be the reasons for these disparities and affect sexuality- and gender-diverse young people and their families from early life.^29,38,44,45^ Sexuality- and gender-diverse young people also experience barriers to health care access, including discrimination and noninclusive practices.^3,4,42,46,47,48,49,50^ A better understanding of developmental trajectories, based on longitudinal studies using nuanced questions, will help to inform health services and support clinical decision-making.

Our estimates of sexuality diversity in adulthood (12.6% of cisgender women and 9.6% of cisgender men) are higher than those reported in Australian population surveys for those aged 25 to 34 years (4.6% of women and 5.2% of men).^51^ Our data are more recent than the surveys, and our participants are younger; in these surveys, sexuality diversity was most prevalent in the most recent cohorts and youngest age groups. Similarly, in a nationally representative sample of Australians aged 16 to 69 years, from 2012 to 2013, 3.6% of women and 3.3% of men identified as sexuality diverse, 14.8% and 6.9% were same-gender attracted, and 13.8% and 6.6% reported lifetime same-gender sexual behavior,^52^ significantly higher than in the 2001 to 2002 study iteration.^52^ In the 2012 to 2013 survey, 21.4% of women and 7.3% of men aged 20 to 29 years reported lifetime same-gender sexual behavior,^53^ comparable to our estimates of 18.6% of women and 7.2% of men.

There are no recent age-specific, population-based estimates of gender diversity in young adulthood in Australia. In a nationally representative survey of secondary school students younger than our participants, 2.3% identified as transgender or gender diverse.^6^ Our estimate of gender diversity, 0.87%, is slightly higher than the 0.6% of Americans aged 25 to 64 years^11^ and 0.51% of Canadians aged 25 to 40 years.^54^ We used a variant of the 2-step method, which separately ascertains sex recorded at birth and current gender and then combines them. A recent meta-analysis found that using this method results in a range of prevalence from 1.7% to 3.1% in cohorts aged 30, 27, and 23 years.^10^

Many more children and adolescents endorsed the ASEBA items than the 10 individuals who identified as transgender or gender diverse. As a result of these findings, we strongly suggest that researchers do not continue to use these ASEBA items as an index of gender diversity. Asking direct questions about gender will provide much more accurate data. Our findings suggest that the ASEBA gender role measures may capture the experience of diverse sexual orientation for some in young adulthood. This association began in childhood and suggests that diverse sexual orientation begins similarly early. For more than 2 decades, qualitative research has shown that young children have a strong awareness of gender and sexuality diversity from approximately 3 years of age.^55,56,57,58,59,60^ Children in familial and social settings surveil and regulate their peers’ gender and sexuality. Simultaneously, they adapt their own gender expression and sexuality to meet dominant social norms and expectations (eg, cisgender and heterosexual).^61,62^

In the quantitative literature, the previous study using ASEBA measures,^20^ and another prospective study using parent-reported gender-typed behavior (Preschool Activities Inventory),^63^ found that relationships to diverse sexual orientation begin at a similarly early age. Although some milestones of developing a diverse sexual identity, such as age at first same-gender sexual attraction, may begin as late as adolescence,^64^ this does not preclude the existence of earlier behavioral characteristics common in children who become sexuality-diverse adults. Still, these measures should not be used to infer sexual orientation in clinical or research settings. The development of diverse sexual orientation in adulthood has many other pathways, and the ASEBA items in childhood are neither sensitive nor specific for this life experience. A previous study compared CBCL gender role wish to parent-reported gender-typed behavior (Gender Identity Questionnaire for Children) in a convenience sample aged 6-12 years. In that study, CBCL showed poor sensitivity.^65^ However, both ASEBA measures have been used in the research literature to define gender diversity, particularly to consider associations between gender diversity and mental health^18,19,66^ or autism^16,67,68,69^ in large, population-based studies; our findings show that specific, validated measures should be used instead for both sexuality and gender diversity.

### Limitations

Limitations of the current study include the fact that gender identity was measured only in adulthood. Sexual behavior has dimensions beyond partner gender, including sexual acts and partner body parts, which are not addressed in the current study. Measures conflated gender and sex by confusing the designations of sex (male and female) with the binary gender designations (man/boy and woman/girl). Measures used for sexual attraction were limited by the assumption of binary gender (“same” and “opposite” sex) on the part of both the respondent and people to whom the respondent is attracted. Because of this and small numbers, we were able to look at dimensions of sexual orientation only among cisgender people. Additionally, the Raine Study Gen2 cohort has been subject to approximately 60% attrition, comparable to other large birth cohort studies (eg, Avon Longitudinal Study of Parents and Children).^70^ Because each follow-up necessarily contains a slightly different subset of the original sample, each follow-up is affected by different combinations of (measured and unmeasured) confounders, and this should be taken into account in interpreting comparisons between follow-ups. Although participating in childhood or adolescent waves was associated with year 27 follow-up participation, there was no association between completion of or response to ASEBA items, at any follow-up, and year 27 participation. This finding reassures us that sexuality- and gender-diverse people were unlikely to selectively drop out.

## Conclusions

This cohort study’s findings indicate that ASEBA items 5 and 110, which have been used to measure gender diversity in childhood in the past, do not reflect gender identity and, despite the associations seen, should not be used to infer sexual orientation. Some gender-diverse people become aware of their gender identity in early childhood, which may remain consistent for them over time.^71,72^ Because gender is an internal construct, we should ask the child directly about their gender. Children self-report on surveys from primary school age, and neurotypical children are aware of their gender by 3 years of age.^71,72,73^ More accurate parent-report measures could also be developed, informed by recent work in Australia^27^ and elsewhere,^74^ to capture accurate gender identity (sex recorded at birth and current gender identity in a 2-step process).^10^

However, it is clear that gender role nonconformity occurs from early childhood, regardless of whether it is linked to later sexuality or gender diversity. Transgressions from cisgender and heteronormative behaviors expose children and adolescents to stigma and bullying, which may account for the previously identified associations between these items and poorer mental health. For 3 decades, qualitative studies in the social sciences have demonstrated the negative impact of homophobic and transphobic harassment and violence experienced by children who transgress gender norms, which affects their health and educational outcomes.^75,76,77,78,79,80,81^ To support the health and well-being of sexuality- and gender-diverse young people, further quantitative research is warranted to understand the mechanisms of this association.

## Notes

Supplement 1. **eTable 1.** Comparison to Population-Based Data **eTable 2.** Relationships Between Completion of ASEBA Items in Previous Follow-ups and Gen2 Participant Attendance at Year-27 Follow-up

Supplement 2.
Data Sharing Statement
