# Supplementary material for: Childhood and Adolescence Gender Role Nonconformity and Gender and Sexuality Diversity in Young Adulthood
Source: JAMA Pediatr. 2023 Sep 25;177(11):1176–86. doi: 10.1001/jamapediatrics.2023.3873 (PMC10520839; doi:10.1001/jamapediatrics.2023.3873)
Supplement: Supplement 1. — eTable 1. Comparison to Population-Based Data eTable 2. Relationships Between Completion of ASEBA Items in Previous Follow-ups and Gen2 Participant Attendance at Year-27 Follow-up [file jamapediatr-e233873-s001.pdf]

Supplementary Online Content

Marino JL, Lin A, Davies C, Kang M, Bista S, Skinner SR. Measures of childhood and adolescence gender role nonconformity and gender and sexuality diversity in young adulthood. *JAMA Pediatrics.* Published online September 25, 2023.
doi:10.1001/jamapediatrics.2023.3873

eTable 1. Comparison to Population-Based Data

eTable 2. Relationships Between Completion of ASEBA Items in Previous Follow-ups and Gen2 Participant Attendance at Year-27 Follow-up

This supplementary material has been provided by the authors to give readers additional information about their work.

© 2023 Marino JL et al. *JAMA Pediatrics.*

eTable 1. Comparison to population-based data

	Western Australians 25-29 years old %	Gen2 participants of the Raine Study Year 27 %
Employment status		
Full time	49.4	57.0
Part time	17.3	19.2
Employed, away from work	4.2	4.0
Unemployed, out of labor force	20.9	5.6
Other [a]	-	9.4
Missing, unstated, unknown	8.2	4.8
Total personal income, weekly		
<$1	7.8	4.8
$1-$299	7.7	8.5
$300-399	4.2	5.5
$400-799	18.7	16.9
$800-999	10.5	12.8
$1000-1249	12.5	19.6
$1250-1449	9.4	11.4
$1500-1999	11.4	12.8
$2000-2999	5.9	6.2
$3000+	1.8	0.8
Missing, unstated	10.0	0.8

a. This category is not included in 2016 Census Labour Force Status classification, and includes casual work, unpaid/voluntary work, self-employment, home/carer duties, and unspecified work.

© 2023 Marino JL et al. *JAMA Pediatrics.*

eTable 2. Relationships between completion of ASEBA items in previous follow-ups and Gen2 participant attendance at year-27 follow-up

	**Year-27 follow-up**		
	**Did not attend N=1714 N (%)**	**Attended N=1154 N (%)**	***p***
Year 5			
Did not attend follow-up	570 (33.3)	98 (8.5)	**<0.001**
Skipped CBCL	17 (1.0)	3 (0.3)	0.2
Skipped ASEBA gender behavior item	9 (0.5)	15 (1.3)	**0.04**
Skipped ASEBA gender wish item	9 (0.5)	7 (0.6)	0.8
Skipped both	1 (0.1)	1 (0.1)	>0.9
Non-conforming ASEBA gender behavior	121 (10.9)	127 (12.3)	0.5
Non-conforming ASEBA gender wish	25 (2.2)	21 (2.0)	0.7
Year 8			
Did not attend follow-up	660 (38.5)	95 (8.2)	**<0.001**
Skipped CBCL	12 (0.7)	13 (1.1)	0.3
Skipped ASEBA gender behavior item	8 (0.5)	4 (0.4)	0.8
Skipped ASEBA gender wish item	8 (0.5)	5 (0.4)	>0.9
Skipped both	0	0	-
Non-conforming ASEBA gender behavior	85 (8.3)	96 (9.2)	0.4
Non-conforming ASEBA gender wish	17 (1.6)	22 (2.1)	0.4
Year 10			
Did not attend follow-up	751 (43.8)	93 (8.1)	**<0.001**
Skipped CBCL	5 (0.3)	0	0.09
Skipped ASEBA gender behavior item	4 (0.2)	2 (0.2)	>0.9
Skipped ASEBA gender wish item	1 (0.1)	2 (0.2)	0.6
Skipped both	0	0	-
Non-conforming ASEBA gender behavior	48 (5.1)	55 (5.2)	0.9
Non-conforming ASEBA gender wish	11 (1.2)	9 (0.9)	0.5
Year 14 – Gen1 parent			
Did not attend follow-up	923 (53.9)	146 (12.7)	**<0.001**
Skipped CBCL	9 (0.5)	3 (0.3)	0.4
Skipped ASEBA gender behavior item	1 (0.1)	4 (0.4)	0.2
Skipped ASEBA gender wish item	1 (0.1)	1 (0.1)	>0.9
Skipped both	0	0	-
Non-conforming ASEBA gender behavior	29 (3.7)	31 (3.1)	0.5
Non-conforming ASEBA gender wish	3 (0.4)	6 (0.6)	0.5
Year 17 – Gen1 parent			
Did not attend follow-up	1175 (68.6)	280 (24.3)	**<0.001**
Skipped CBCL	14 (0.8)	10 (0.9)	>0.9
Skipped ASEBA gender behavior item	2 (0.1)	0	0.5
Skipped ASEBA gender wish item	1 (0.1)	4 (0.4)	0.2
Skipped both	0	0	-
Non-conforming ASEBA gender behavior	14 (2.7)	22 (2.6)	0.9
Non-conforming ASEBA gender wish	3 (0.6)	4 (0.5)	0.8
Year 14 – Gen2 adolescent			
Did not attend follow-up	1049 (61.2)	214 (18.5)	**<0.001**
Skipped CBCL	6 (0.4)	2 (0.2)	0.5
Skipped ASEBA gender wish item	1 (0.1)	0	>0.9
Non-conforming ASEBA gender wish	41 (6.2)	42 (4.5)	0.1
Year 17 – Gen2 adolescent			
Did not attend follow-up	394 (23.0)	855 (75.1)	**<0.001**
Skipped CBCL	1 (0.1)	2 (0.2)	0.6
Skipped ASEBA gender wish item	4 (0.2)	13 (1.1)	**0.005**
Non-conforming ASEBA gender wish	37 (9.5)	82 (9.8)	0.9

ASEBA: Achenbach System of Empirically Based Assessment

© 2023 Marino JL et al. *JAMA Pediatrics.*
