# Supplementary material for: Childhood and Adolescence Gender Role Nonconformity and Gender and Sexuality Diversity in Young Adulthood
Source: JAMA Pediatr. 2023 Sep 25;177(11):1176–86. doi: 10.1001/jamapediatrics.2023.3873 (PMC10520839; doi:10.1001/jamapediatrics.2023.3873)
Supplement: Supplement 2. — Data Sharing Statement [file jamapediatr-e233873-s002.pdf]

Data Sharing Statement

Marino. Childhood and Adolescence Gender Role Nonconformity and Gender and Sexuality Diversity in Young Adulthood. *JAMA Pediatr*. Published September 25, 2023. doi:10.1001/jamapediatrics.2023.3873

Data
Data available: No

Additional Information
Explanation for why data not available: Data are not publicly available due to the terms of ethics approvals granted by UWA and Curtin University HRECs and data disclosure policies of the Raine Study. Data may be available from the corresponding author upon request and subject to approval from the UWA and Curtin University HRECs and the Raine Study. Data may be accessed by contacting the corresponding and senior authors (JLM and SRS, respectively) for approval to join the author group, then following the Raine Study Research Engagement Policy, available at the Raine Study website (rainestudy.org.au).
